# Probing femtosecond lattice displacement upon photo-carrier generation in lead halide perovskite

**DOI:** 10.1038/s41467-018-04367-6

**Published:** 2018-05-17

**Authors:** Giovanni Batignani, Giuseppe Fumero, Ajay Ram Srimath Kandada, Giulio Cerullo, Marina Gandini, Carino Ferrante, Annamaria Petrozza, Tullio Scopigno

**Affiliations:** 1 Dipartimento di Fisica, Università di Roma “La Sapienza”, Roma, I-00185 Italy; 2 Dipartimento di Scienze di Base e Applicate per l’Ingegneria, Università di Roma “La Sapienza”, Roma, I-00185 Italy; 3 Center for Nano Science and Technology @Polimi, Istituto Italiano di Tecnologia, via Giovanni Pascoli 70/3, 20133 Milan, Italy; 4 Dipartimento di Fisica, Politecnico di Milano, Piazza L. da Vinci, 32, 20133 Milano, Italy; 5 Istituto Italiano di Tecnologia, Center for Life Nano Science @Sapienza, Roma, I-00161 Italy

## Abstract

Electronic properties and lattice vibrations are expected to be strongly correlated in metal-halide perovskites, due to the soft fluctuating nature of their crystal lattice. Thus, unveiling electron–phonon coupling dynamics upon ultrafast photoexcitation is necessary for understanding the optoelectronic behavior of the semiconductor. Here, we use impulsive vibrational spectroscopy to reveal vibrational modes of methylammonium lead-bromide perovskite under electronically resonant and non-resonant conditions. We identify two excited state coherent phonons at 89 and 106 cm^−1^, whose phases reveal a shift of the potential energy minimum upon ultrafast photocarrier generation. This indicates the transition to a new geometry, reached after approximately 90 fs, and fully equilibrated within the phonons lifetime of about 1 ps. Our results unambiguously prove that these modes drive the crystalline distortion occurring upon photo-excitation, demonstrating the presence of polaronic effects.

## Abstract (web summary)

The electron–phonon coupling is the key to understand optoelectronic properties in lead halide perovskites but it is difficult to probe. Here Batignani et al. observe two new phonon modes with impulsive vibrational spectroscopy providing the evidence of the polaronic nature of the photo-excitation.

## Introduction

Solution processed hybrid lead-halide perovskites are an emergent class of materials for efficient optoelectronic devices^1^. Despite the technological appeal, a comprehensive understanding of their photo-excitation dynamics is still lacking. A crucial, missing information is the nature of carriers, a key issue for the description of transport and recombination processes, two unique characteristics of these materials also in focus of a lively debate^2–4,5,6^. In fact, the reported carrier recombination rates^7^ are remarkably low, i.e., comparable to the best ones reported for single crystalline semiconductors^2^ and orders of magnitude lower than those predicted by the Langevin model^4^. On the other hand, modest charge mobilities have been reported, i.e., much lower than those of crystalline semiconductors albeit higher than those found in organic (disordered) semiconductors^8^. Given the polar nature of the perovskite lattice, it has been suggested^8^, in order to justify the low mobilities, that carriers localize as large polarons in contrast to a pure band-like picture where carriers act as delocalized Bloch waves. This picture would be consistent with carrier transport and temperature dependence of the homogenous linewidths of electronic transitions^9^, pointing to the presence of strong electron–phonon scattering mechanism. The importance of this interaction mechanism has also been indicated by light-enhanced local disorder observed through electron diffraction^10^. Recently, it has been suggested^11^ that photogenerated electrons relax into a distinct dark electronic state which extends the charge carrier lifetime. Although the proposed mechanism is compatible with both the band-like and large polaron pictures, an experimental method able to disentangle these effects is still missing. Therefore, both the nature of such state and the reaction pathway which would lead to its population remain unclear^12^.

Here, we use impulsive vibrational spectroscopy (IVS), a time domain Raman technique, to unveil the phonon spectra of the ground and excited electronic states in methylammonium lead-bromide perovskite (MAPbBr_3_) polycrystalline thin films. Raman spectroscopy is an ideal tool to investigate electron–phonon coupling in materials^13,14^. While ground state vibrational features in lead halide perovskites have been thoroughly addressed^15–20^, identifying the correlation between the phonon modes and electronic excitations is one of the major challenges in the field. To address this issue, it would be imperative to perform the measurements with a resonant excitation. Detecting spontaneous Raman signals from excited electronic states using a single resonant excitation is not trivial. Moreover, the presence of a large background signal from photoluminescence (PL) may obscure the low frequency region that contains the most relevant modes of the inorganic moiety. Within this context, IVS^21–23^ represents a powerful technique to circumvent these limitations and resolve both ground and excited state Raman bands with high spectral resolution. In fact, since the measurement is performed in the time domain, there are no spectral limitations neither artefacts arising from elastic pump-scattering and PL.

**Fig. 1:**
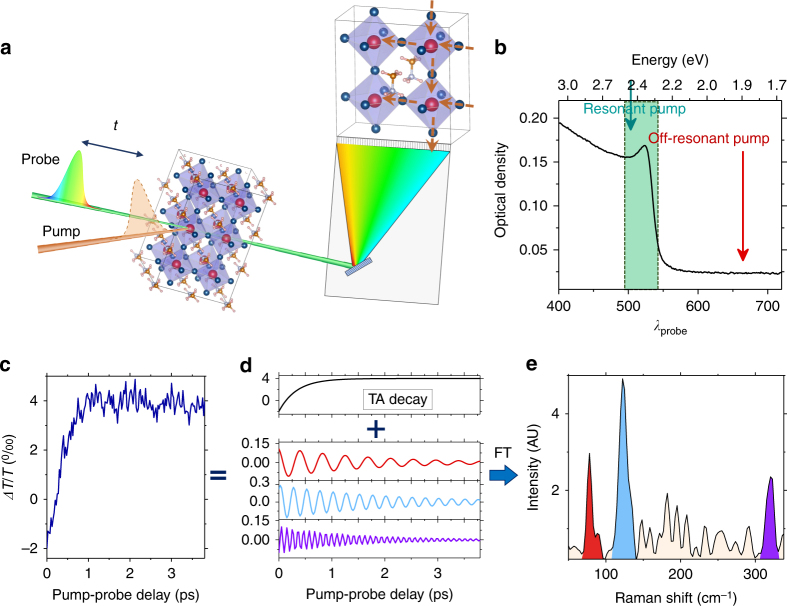
Concept of the Impulsive vibrational spectroscopy experiment on MAPbBr_3_. **a** IVS pulse scheme and MAPbBr_3_ crystal structure. After a tunable delay from the interaction with a femtosecond pump pulse, an ultrashort broadband probe pulse interrogates the system and reveals the stimulated lattice vibrations. **b** Visible absorption spectrum of a polycrystalline thin film of MAPbBr_3_: the red and green arrows indicate respectively the 1.86 eV off-resonant and the resonant 2.46 eV pump photon energies used in our experiments. The colored box represents the analyzed probe spectral regions. **c** The experimentally detected differential signal shows the photo-induced modifications of the transmission profile as a function of the time delay between the two pulses. **d** The signal consists in oscillating components, which carry the phonon frequencies, superimposed to the TA exponential dynamics. **e** The vibrational information is directly obtained by Fourier Transforming the experimental data after the subtraction of the TA decay

IVS exploits two femtosecond laser pulses to obtain the Raman vibrations of the system under investigation (Fig. 1a). First, Raman interactions convert an incoming optical pump pulse into an impulsive force acting on the solid-state lattice. This results in collective lattice displacements, namely coherent phonons, provided that the duration of the pump pulse is shorter than the Raman active mode vibrational period. Then, the interaction with a delayed probe pulse leads to the generation of a third order electronic polarization in the material, which modulates the transmission of the probe. The overall result is the appearance of a time oscillating transmissivity change at the frequencies of the stimulated coherent phonon modes, detected by the time-delayed probe pulse. This oscillating response is always superimposed on a transient absorption (TA) signal, which arises when carriers are photo-generated. The underlying exponential electronic kinetics responsible for such TA background is subtracted from the detected signal to extract the oscillating temporal dynamics^22,24^. These time domain signals are then Fourier transformed to obtain the vibrational Raman spectra. This allows us to determine the phonons coupled to the electronic transition that rule the structural rearrangement in MAPbBr_3_ upon photo-carrier generation.

## Results

**Fig. 2:**
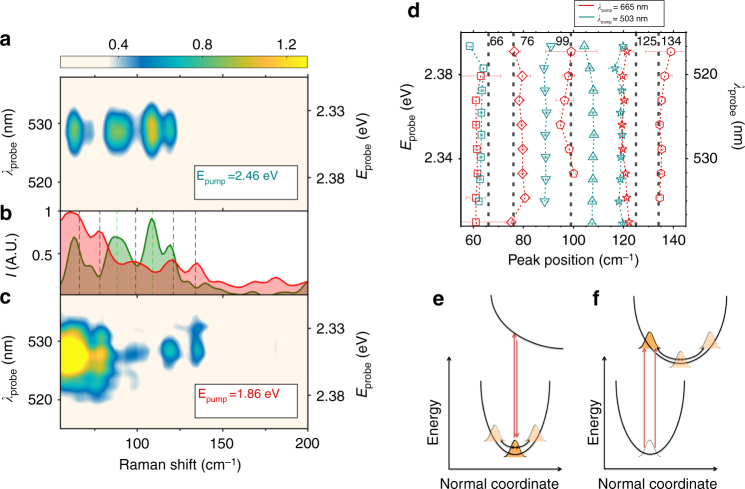
Impulsive vibrational spectroscopy on MAPbBr_3_ under different resonance regimes. Probe-wavelength resolved IVS maps, upon resonant (*E*_Pump_ = 2.46 eV) and non-resonant (*E*_Pump_ = 1.86 eV) excitations (**a**, **c**, respectively). The maps have been obtained by Fourier transforming the oscillating component of the TA data (see Fig. 1), to retrieve the vibrational spectra. **b** Average of the IVS maps over different probe wavelengths. Green and red shaded Raman spectra refer to resonant and non-resonant conditions, respectively. **d** Fitted peaks positions of the measured Raman modes (with the corresponding 95% confidence boundaries) as a function of the probed wavelength. Red and green symbols refer to Raman modes obtained by pumping at 1.86 and 2.46 eV, respectively. Vertical dashed lines indicate the position of MAPbBr_3_ ground state vibrational modes reported in literature^25^. **e**, **f** Representations of the ISRS and DECP processes, respectively. The red arrows indicate the double interactions with the pump pulse, which generates a vibrational coherence in the ground and excited state^27^. In ISRS, immediately after photoexcitation, the vibrational wavepacket is peaked at the equilibrium position and then starts oscillating along the normal mode coordinate. In DECP, the ground state wavepacket is projected onto the excited state, where it begins oscillating from a starting position far from the equilibrium. We stress that for other class of materials, as III–V semiconductors, additional generation mechanisms are possible^42^

Figure 1b shows the absorption spectrum of the polycrystalline thin film of MAPbBr_3_ (see Methods section for details on sample preparation), where the red and green arrows indicate the 1.86 eV (665 nm) off-resonant and 2.46 eV (503 nm) resonant excitation pulses used in our experiments. To avoid photo-degradation, the sample was encapsulated by a thick PMMA layer and measured in air, preventing the formation of intra-gap defect states^25^. The basics of the performed IVS experiment are provided in Fig. 1c–e and detailed in Supplementary Note 1. For both the actinic excitation energies, we observe an underlying transient absorption signal, in agreement with previous reports^26^. The pump pulse duration is 30 (50) fs for the 1.86 (2.46) eV, which is much shorter than the periods of the vibrational modes of interest. Figure 2a–c reports the IVS spectra under such excitations. Since the detection of the probe is spectrally dispersed, it provides us with a probe-wavelength resolved vibrational coherence map^27–29^. The exact procedure used for the data analysis is detailed in Supplementary Note 1. Briefly, for both the off-resonance and resonant excitations, we consider probe wavelengths around the TA maximum (from 510 to 540 nm, photon energies from 2.43 to 2.30 eV), where the IVS cross section is enhanced. Multiple TA traces acquired during the experiment are reported in Supplementary Figure 4. To factor out cross phase modulation artefacts, due to pump and probe temporal overlap, time traces have only been considered from 250 fs onwards in the data analysis. After removing the superimposed dynamics from the TA signal (Supplementary Figure 2), we apply Fourier transformation at each probe wavelength. Zero padding algorithm and Kaiser–Bessel windowing are exploited to enhance the spectral definition. The resulting 2D vibrational maps are shown in Fig. 2a and 2c. The average of these maps over all the detected probe wavelengths is reported in Fig. 2b (while slices at selected wavelengths are shown in Supplementary Figure 3), where the green and red shaded spectra correspond to resonant and off-resonant excitations, respectively. The obtained Raman spectra are fitted with Gaussian functions to extract the peak positions of the various modes, which are plotted as a function of probe wavelength in Fig. 2d, for both the pumps. The 2D vibrational map recorded at red-shifted probe wavelengths are reported in Supplementary Figure 5.

In the off-resonant case, we observe peaks at 64, 78, 98, 121, 134 cm^−1^. These frequencies are in good agreement with theoretical calculations and continuous wave (CW) Raman spectra^18,30,31^ (for which the peak positions of the modes are reported in Fig. 2d as dashed vertical lines^31^), and represent the vibrational degrees of freedom exhibited by the lead-halide octahedra, pointing to the inherent softness of the inorganic cage^15^. The modes at 98 and 134 cm^−1^ have been assigned to the symmetric stretch modes of the Br–Pb–Br bond, the mode at 121 cm^−1^ to the asymmetric stretch. The 64 and 78 cm^−1^ are the corresponding symmetric and asymmetric scissoring modes. The measured full widths at half maximum of the modes are in agreement with phonon dephasing times *Γ*^−1^ ~1 ps. Under such non-resonant excitation regime, coherent phonons are generated exclusively by a Stimulated Raman Scattering process, Impulsively activated by a pump pulse shorter than the phonon period (ISRS)^21^. Within such a scenario, coherent lattice motions are induced in the electronic ground state and the obtained spectrum is analogous to the CW Raman.

Using green resonant pump, we observe four modes of which 64 and 121 cm^−1^ were also present in the non-resonant case. The strong presence of these modes in the current case can be attributed to the selective enhancement of the ISRS for the ground state vibrational modes which are strongly coupled to the electronic transition^32,33^ via electron–phonon interactions (Fig. 2e). Such coupling has also been observed via temperature dependent photoluminescence spectroscopy^9^ and via mid infrared spectroscopy^34^. Remarkably, we observe additional phonon modes at 89 and 106 cm^−1^ that are not present in the CW Raman. In fact, in the presence of an excited state potential energy surface shifted with respect to the ground state, coherent vibrational modes can also be stimulated via a displacive excitation process (DECP). This is illustrated in Fig. 2f for the case of molecular systems, where a geometrical rearrangement of the atoms is driven by the dynamics of the vibrational wavepacket in the electronically excited state. In close analogy, in crystalline materials DECP allows for the generation of coherent phonons at frequencies distinct from the ground state vibrational modes^35^. Hence, we suggest the 89 and 106 cm^−1^ lines, appearing only in resonant-IVS, to be DECP generated. Analysis of the fluence dependence and phase lag between the measured phonons (presented in Supplementary Note 2) further substantiate this assignment. Specifically: (i) ISRS and DECP modes scale differently with pump fluences (Supplementary Figure 7). (ii) The relative phase difference between those modes observed in the non-resonant case is approximately null (see Supplementary Note 2 and Supplementary Figure 6), due to a wavepacket generation occurring in the energy minimum of the stimulated vibrational coordinate (Fig. 2e). On the other hand, there is a non-zero phase difference between the ISRS modes (64 and 121 cm^−1^) and the DECP (89 and 106 cm^−1^) observed in the resonant-IVS (Supplementary Figure 6), as these latter originate from the vertical projection of the initial ground state wavepacket onto an out-of-equilibrium position of the new lattice geometry (Fig. 2f). Accordingly, these are the modes driving the crystalline distortion through transient lattice oscillations, leading to a new configuration—along a specific reaction coordinate—reached after a quarter of the period of the corresponding phonon (*T*/4 = 80 and *T*/4 = 95 fs), and completely relaxed within the phonon lifetime (*Γ*^−1^ ~1 ps).

## Discussion

Although in crystalline materials the lattice coordinates of the excited states are usually expected to remain similar to that of the ground state^36^, our observations indicate a geometrical reorganization upon electronic excitation in the case of methylammonium lead-bromide perovskite, which may be due to their strongly polar nature^37^. This is consistent with some recent observations on hybrid perovskites performed by means of optical Kerr effect [7]. Notably, shifts in the equilibrium positions and vibrational energies of the crystal in the presence of photo-generated carriers presented here are highly suggestive of strong electron–phonon coupling and thus of the polaronic nature of the carriers^38^. Within the Fröhlich model^39^ (see Supplementary Note 3), an estimate of the electron–phonon coupling strength is expressed through the dimensionless coupling constant *α*^38^, which can be directly extracted from the DECP phonon frequencies obtained by the IVS measurements as

1 $$\alpha = \frac{{e^2}}{\hbar }\frac{1}{{4\pi {\it{\epsilon }}}}\sqrt {\frac{{m^ \ast }}{{2\hbar \omega _{{\mathrm{eff}}}}}} \left( {\frac{1}{{\varepsilon _\infty }} - \frac{1}{{\varepsilon _0}}} \right)$$

*ω*_*eff*_ represents the weighted sum of the DECP frequencies and is calculated through the relation $\omega _{eff} = \sqrt {\mathop {\sum}\nolimits_i {A_i^2/\mathop {\sum }\limits_i \frac{{A_i^2}}{{\omega _i^2}}} }$, being *A*_*i*_ the spectral amplitudes of the modes, *ε* is the dielectric constant of vacuum, while we consider *ε*_0_ = 25 and *ε*_∞_ = 6.7 for the static and high frequency dielectric constants^40^. We estimate *α*_e_ = 1.84, *α*_h_ = 1.28, for electron and hole, respectively. We note that these values are in agreement with those obtained for other hybrid perovskites by previous studies^9^, in which the estimations were based on combined first-principles calculations and indirect PL measurements.

These results allow estimating a lower bound for the polaron binding energy (*E*_b_), which can be evaluated from the DECP frequencies and a perturbative expansion in terms of the coupling constant α within the Fröhlich formalism, obtaining an effective value $\frac{{E_{\mathrm b}}}{{K_{\mathrm B}}} \approx {{538}}$ K. This suggests a possible rationale for the extended carrier lifetime. Indeed an energy barrier equal to *E*_b_, significantly above the thermal energy *k*_B_*T*, should be crossed by the carriers to relax back into the groundstate and restore the original lattice geometry.

It should be stressed that our estimates are based on electron–phonon interactions within a Fröhlich-like mechanism, whilst the observation of lattice reorganization upon photo-excitation suggests presence of additional strong coupling contributions. The unambiguous ultrafast dynamics presented here can offer experimental benchmarks in order to further develop polaron models in these materials.

Interestingly, on the timescale of the observed lattice rearrangement, the transient absorption spectrum presents a photo-bleaching band arising from the state-filling convolved with a derivative like line-shape (see Supplementary Figure 1), which is generally the signature of Coulomb effects on the electronic/excitonic transition^41^. Although photo-bleaching follows the charge carrier dynamics, living for hundreds of picoseconds^26^, the derivative feature decays within the first picosecond, i.e., during the time interval taken by the new lattice geometry to equilibrate, supporting the presence of ionic displacements which could potentially screen any Coulomb correlations.

In conclusion, by contrasting the response obtained upon resonant and non-resonant pumping, we demonstrated how IVS can be employed to isolate those phonons coupled to a specific electronic transition in hybrid perovskites. Most importantly, we revealed the key phonon modes, generated via displacive excitation mechanism, which provide the pathway to the photo-induced lattice modification occurring upon carrier generation. Since electronic band states, as well as Wannier excitons would not show an associated pattern of displaced atomic equilibrium positions, our results provide evidence for the polaronic nature of photo-excitation in methylammonium lead-bromide perovskite. We anticipate our approach to be applicable to different lead halide perovskites to reveal general aspects and system-specific peculiarities of the photocarriers nature.

## Methods

### Sample preparation

Lead(II) bromide (PbBr_2_, ≥98%) and N,N-dimethylformamide (DMF, anhydrous, 99.8%) were purchased from Sigma-Aldrich; methylammonium bromide (MABr) was purchased from Dyesol. All chemicals were used without any further purification. Glass substrates were cleaned in acetone and isopropyl alcohol for 10 min by sonication. The cleaned glass substrates were treated with Oxygen plasma for 10 min before the perovskite deposition. An equimolar solution of PbBr_2_ and MABr was prepared in DMF (20 wt%) and spin coated on the substrate at 3000 rpm for 60 s, and immediately annealed at 100 °C for 15 min, under inert atmosphere. In order to avoid photo-degradation, the sample was encapsulated by a thick polymethyl methacrylate (PMMA) layer and measured in air, which hampers the formation of intra-gap defect states. The details of the experimental setup are reported in Supplementary Note 1.

### Data availability

All relevant data are available from the authors.

## Electronic supplementary material

Supplementary Information
