# Supplementary material for: Probing femtosecond lattice displacement upon photo-carrier generation in lead halide perovskite
Source: Nat Commun. 2018 May 17;9:1971. doi: 10.1038/s41467-018-04367-6 (PMC5958143; doi:10.1038/s41467-018-04367-6)
Supplement: Supplementary file 1 — Supplementary Information [file 41467_2018_4367_MOESM1_ESM.pdf]

SUPPLEMENTARY INFORMATION

Probing Femtosecond Lattice Displacement upon Photo-carrier generation in Lead Halide Perovskite

Giovanni Batignani et al.

Supplementary Figures

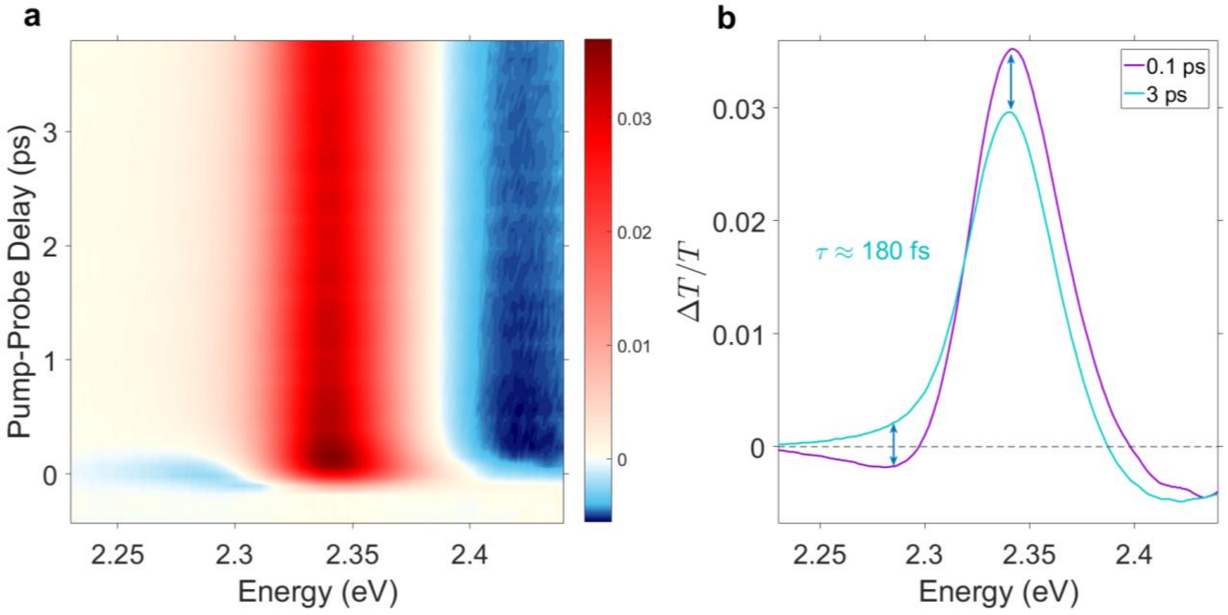

Supplementary Figure 1: Ultrafast response of $MAPbBr_3$ upon a 2.46 eV resonant excitation. ***(a)*** *Differential transmission of $MAPbBr_3$ the as a function of the probe photon energy, showing the first 3.5 ps system response.* ***(b)*** *Selected TA traces at 100 fs (purple line) and 3 ps (blue line) pump-probe time delays.*

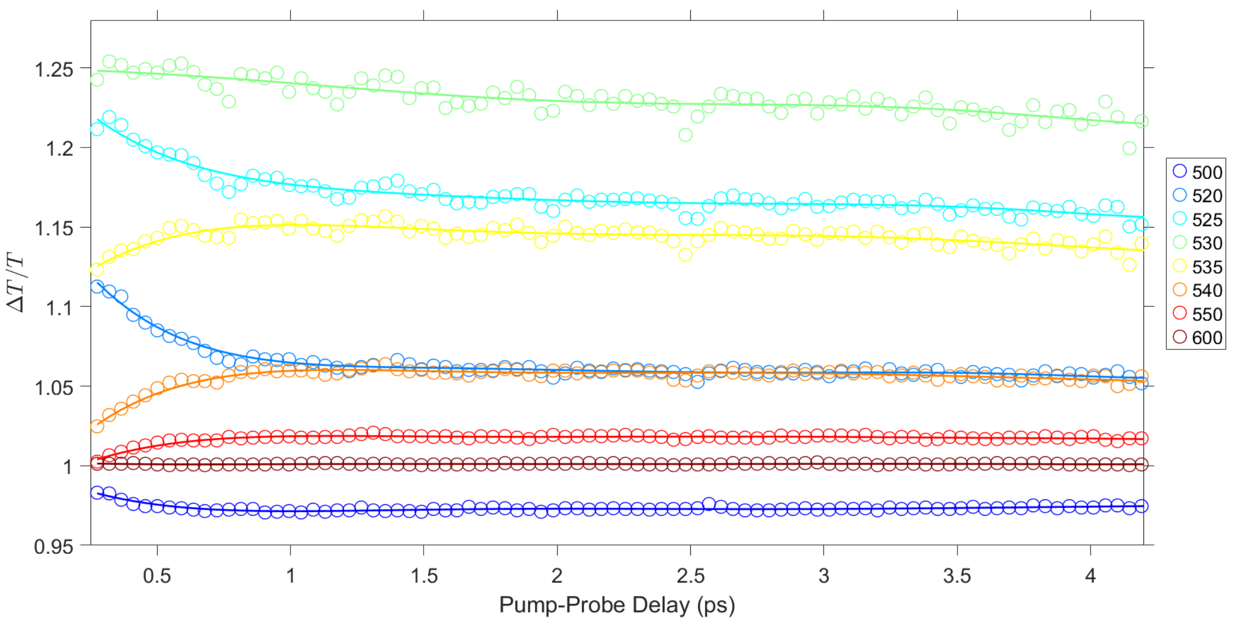

Supplementary Figure 2: IVS traces (dots) before removal of the TA dynamics for selected wavelengths. *Six-order polynomial function, reported as continuous lines, are employed to eliminate the TA dynamics, isolating the vibrational fast oscillating components in the detected*

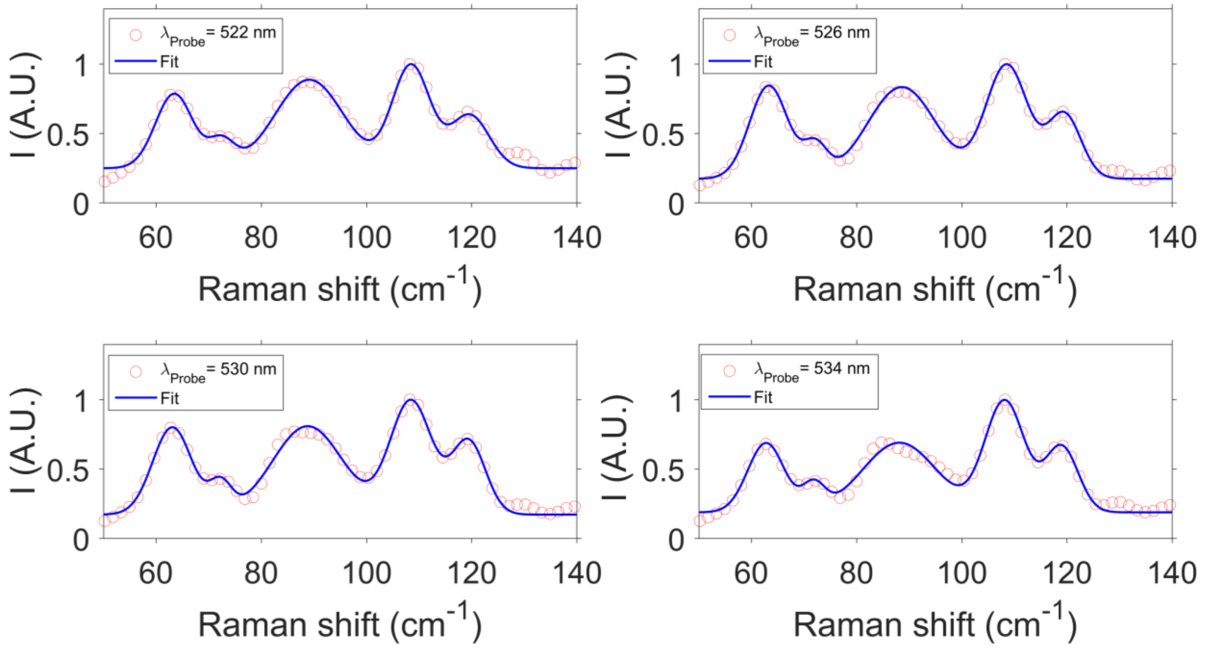

Supplementary Figure 3: Impulsive vibrational spectroscopy measurements of MAPbBr3 upon resonant excitation. *Slices of IVS map, averaged over 4 nm-regions around selected probe wavelengths. The spectral profiles are fitted with the sum of 5 Gaussian profiles (continuous lines) in order to extract the peaks positions of the measured Raman modes.*

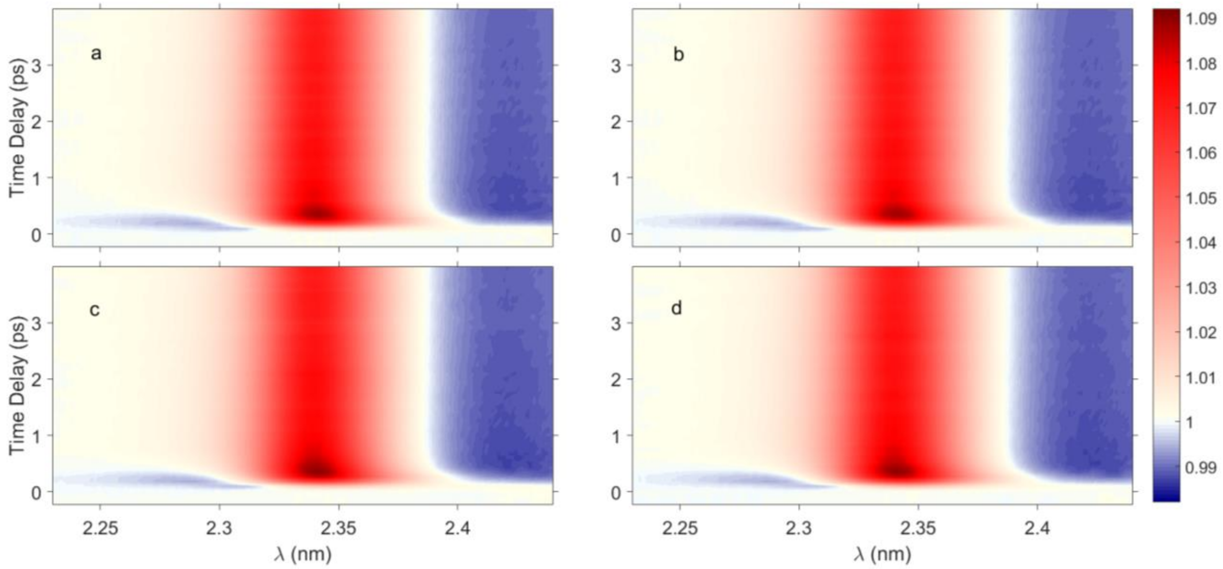

Supplementary Figure 4: multiple TA traces. *Transient absorption maps acquired during the IVS experiment, under resonant photoexcitation regime. Panels a and b report spectra at the beginning of the experiment, while panels c and d at the end.*

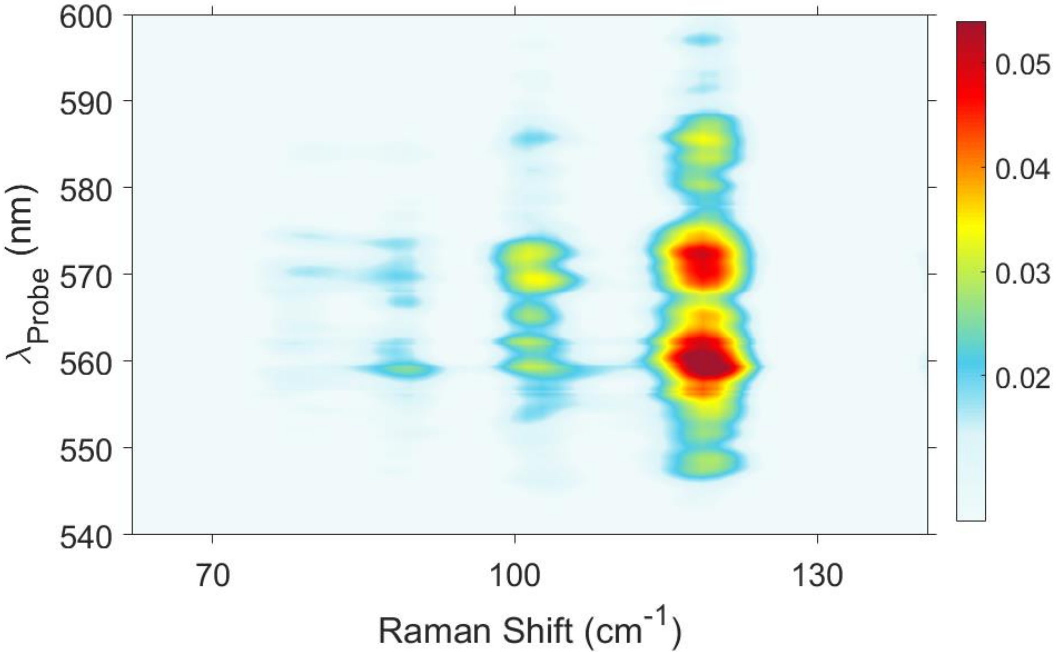

Supplementary Figure 5: IVS spectra for probe wavelengths in the high energy side of the main transition peak. *Probe wavelength resolved IVS map, upon resonant (E_{Pump} = 2.46 eV) photo-excitations in the 540-600 nm probe spectral region.*

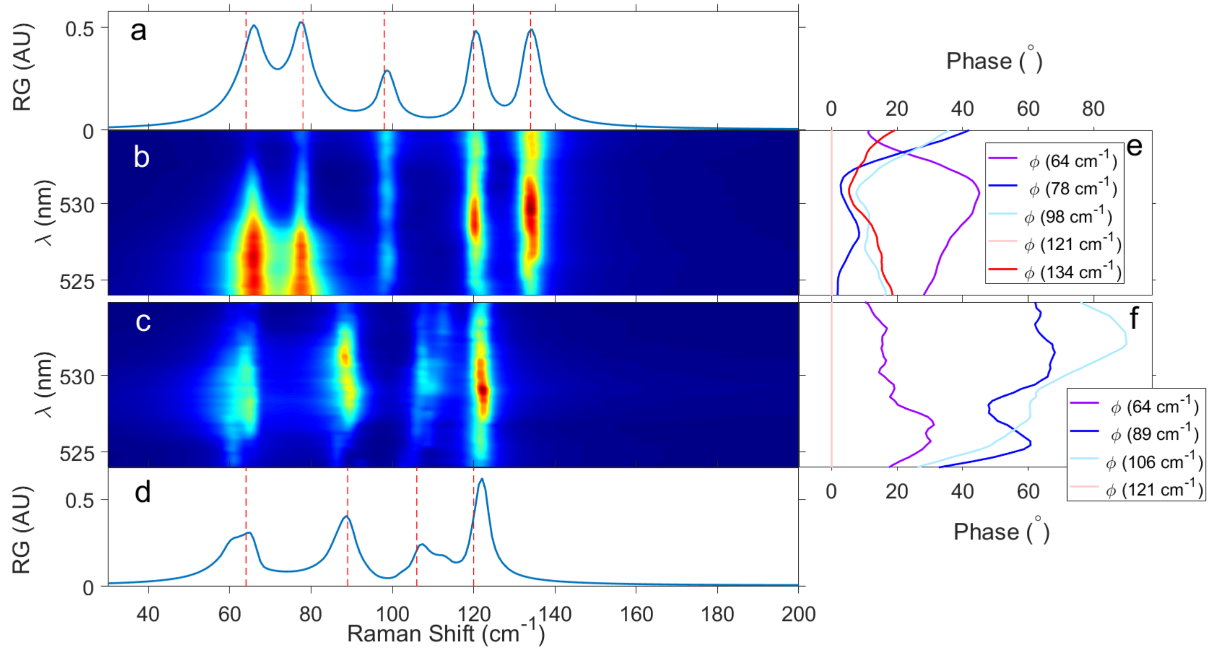

Supplementary Figure 6: Phase Analysis upon non-resonant and resonant photo-excitations. *Panels a and b report the Raman Gain (RG) extracted isolating five oscillating components from the IVS time domain traces under non-resonant condition (integrated and probe wavelength resolved map, respectively), while panel e indicate the relative phases of the phonon modes. In c and d, the Raman Gain (RG) extracted isolating four oscillating components in the IVS time domain traces upon resonant pumping (integrated and probe wavelength resolved map, respectively), while panel f report the relative phases. The vertical dashed lines indicate the average peak position of the Raman line peak position reported in Figure 2b.*

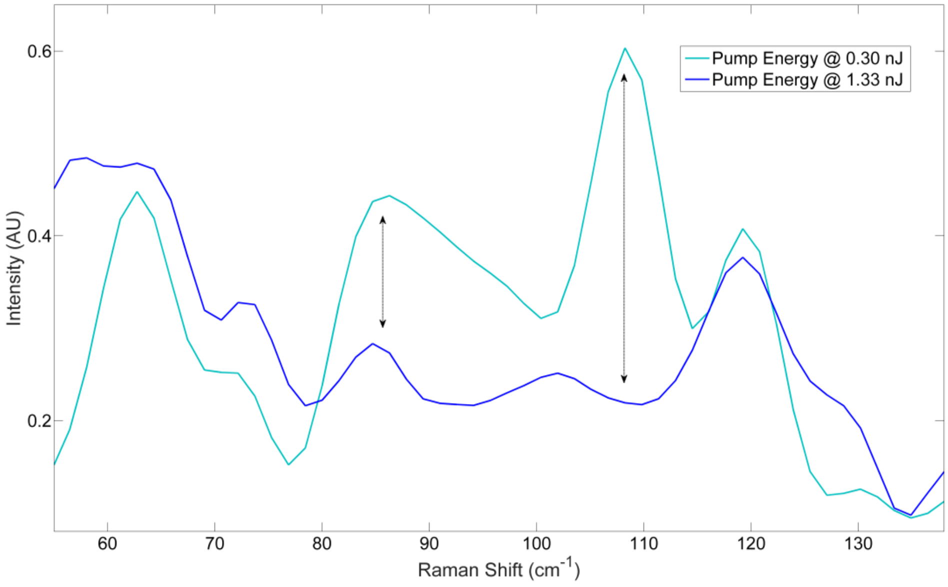

Supplementary Figure 7: IVS spectra upon resonant excitation, measured at two different pump pulse energies. *IVS spectra are normalized at the intensity of the ISRS mode intensities (64 and 121 cm^{-1}). The spectra have been obtained averaging over the detected probe wavelengths. The vertical arrows highlight the different scaling of the DECP phonons (89, 106 cm^{-1}) as a function of the pump energy, pointing to their different nature.*

Supplementary Note 1: Impulsive vibrational spectroscopy experimental details and data analysis.

The experimental setup, developed at the Femtoscopy labs ("Sapienza" University of Rome) and exploited for these measurements, has been further detailed in [1]. It is based on a Ti:sapphire laser source generating 3.6 mJ, 35 fs pulses at 800 nm and 1 kHz repetition rate. The pump pulse is synthesized by a non-collinear optical parametric amplifier (NOPA) that produces tunable visible pulses in the range (480-700 nm). For the off-resonance measurements, $\Delta\lambda_{\text{FWHM}} = 30\ nm, 30\ fs$ pulses centered at 665 nm are generated, while for the resonant ones, $\Delta\lambda_{\text{FWHM}} = 10\ nm, 50\ fs$ pulses centered at 503 nm are produced. The time interval between pump and probe pulses is settled by a computer controlled delay line on the pump pulse optical path. The white light continuum (WLC) probe pulse is synthesized focusing part of the source pulse on a nonlinear medium plate. A synchronized chopper blocks alternating Raman pulses in order to record the modification induced to transmitted WLC probe, which is frequency dispersed by a spectrometer onto a CCD device.

The same experimental setup has been used for both transient absorption (TA) and impulsive vibrational spectroscopy (IVS) measurements. The TA spectrum of $MAPbBr_3$ upon a 2.46 eV resonant excitation is shown in Supplementary Figure 1.

For the IVS experiment, after the interaction with the pump pulse, a pump-probe temporal delay window of 4.25 ps has been analysed, with a 83-fs step. The resulting spectral resolution (Δν) is $\Delta\nu = 7.8\ cm^{-1}$. The same measurements are performed also using time steps of 33 fs and 54 fs and the final (frequency domain) spectra are obtained averaging over the different used time steps. The Actinic fluence on the sample is continuously monitored during the acquisition, in order to correct for pump instability and thus improve the signal to noise ratio.

As shown in Supplementary Figure 2, a global fitting routine, which employs a six-order polynomial function is employed to eliminate the TA dynamics, isolating the vibrational fast oscillating components in the detected spectra. The Raman spectrum is hence obtained from the oscillating temporal signal using fast Fourier transform (FFT) algorithm. In order to increase the frequency produced by the FFT, we lengthen the time window by a factor 3, through the zero-padding algorithm [2]. Moreover, the Raman spectrum is extracted through FFT only after applying a $w(L,\beta)$ Kaiser−Bessel window [3] after the zero-padding procedure.

The Kaiser function $w(L,\beta)$ is equivalent to $J\left(0, \beta \cdot \sqrt{1 - \left\{\frac{\left[n - \frac{N}{2}\right]}{\frac{N}{2}}\right\}^2}\right) \Big/ J(0,\beta)$, where $J(0,z)$ computes the modified Bessel function of the first kind, N indicates the number of acquired temporal delay and n is a vector defined as $n = [0,1,\dots,N]$. We used β=12.

Averages over repeated measures is performed after Fourier transforming the acquired data. Slices of IVS spectra at selected wavelengths, are reported in Supplementary Figure 3. The Raman spectra have been fitted with Gaussian functions in order to extract the peak positions (reported in Fig. 2b). of the various modes.

Notably, acquiring the TA spectra during the IVS measurements enables us to monitor the sample stability. In Supplementary Figure 4, 4 different TA traces, acquired during the IVS measurement upon the resonant photoexcitation, are reported. They do not show any substantial change, clearly indicating the absence of photo-damaging during the measurement.

In Supplementary Figure 5 we report the IVS spectra upon resonant photoexcitation, measured on the low energy side of the main transition peak**.** Despite the lower signal-to-noise ratio and cross section, as expected [1], the modes are also observed in this spectral region.

Supplementary Note 2: DECP and ISRS nature of the detected Raman modes.

As described in the main text, upon photoexcitation, the coherent phonons can be generated by an impulsive stimulated Raman scattering (ISRS) process or a displacive excitation mechanism (DECP). In ISRS, wavepacket oscillations are coherently stimulated in the ground state and undergoes mode-selective resonance enhancement; in DECP, the coherent vibration is generated in the excited state. Since the minimum of the potential energy surface of the excited state is shifted in the coordinate space with respect to that of the ground state, in DECP the wavepacket is initially peaked far from the equilibrium position of the new lattice geometry, which is induced by the photogenerated carriers' distribution. In case of non-resonant excitations, only the ISRS process is possible.

Supplementary Figure 6 illustrates the results of a phase analysis performed on IVS spectra. By using as starting point the frequencies obtained by the FFT described in the main (Fig. 2a-b), the wavelength dispersed time domain traces measured upon non-resonant photoexcitation are decomposed as the sum of five decaying oscillating components, with relative arbitrary phases, amplitudes, peak positions and dephasing times. The results are reported in panels a and b, while the relative phases are shown in panel c and are approximately null. A similar procedure has been performed to decompose the IVS time domain trace as the sum of four components (panels d and e). In this case, as shown in f, we identify a relative phase difference $\approx 50^{\circ}$ between the 89, 106 cm^{-1} and the 64, 121 cm^{-1} modes, indicating that the latter phonons are generated by DECP mechanism.

In order to further corroborate this assignment, we studied the dependence of the IVS phonon modes on the resonant excitation fluence. In Supplementary Figure 7 we compare two measurements performed with different pump energies (0.3 nJ and 1.33 nJ), normalized to the ISRS mode intensities (64, 121 cm^{-1}). Importantly, the 89 and 106 cm^{-1} modes do not follow the same scaling, further testifying to their different nature.

Supplementary Note 3: Fröhlich polaron binding energy.

The properties of polarons within the Fröhlich model are parametrized by the coupling constant α, which has been calculated from the DECP phonon frequencies obtained by the IVS measurements. We stress that, having considered time traces only from 250 fs onwards in the data analysis reduces the confidence interval in the DECP mode relative amplitudes, introducing a 3% uncertainty in the estimate of α.

Fröhlich (1954) has provided the first weak-coupling perturbation theory for calculating the polaron binding energy as

$$E_0 \approx -\alpha\hbar\omega_{TOT} \qquad (1)$$

being ω_{TOT} the sum of the frequencies of the observed DECP phonons. Feynman introduced a variational principle for path integrals to study the polaron, providing an expansion in terms of α for the polaron ground state energy calculation [4]

$$E_F \approx -\hbar\omega_{TOT}[\alpha + 0.0123 \cdot \alpha^2 + 0.00064 \cdot \alpha^3] \qquad (2)$$

More recently, an accurate estimate of the terms in this perturbative expansion has been provided by Selyugin and Smondyrev [5]:

$$E \approx -\hbar\omega_{TOT}[\alpha + 0.0159 \cdot \alpha^2 + 0.00081 \cdot \alpha^3] \qquad (3)$$

Supplementary Equations 1-3 provide estimates of the polaron binding energy which deviates up to 3%.

Numerical studies, investigating finite temperature effect within the Feynman variational approach, point to an increase of the polaron free energy as a function of the temperature. Therefore, our estimate has to be considered as a lower estimate for the polaron binding energy [6].

Supplementary References

[1] L. Monacelli, G. Batignani, G. Fumero, C. Ferrante, S. Mukamel e T. Scopigno, Manipulating Impulsive Stimulated Raman Spectroscopy with a Chirped Probe Pulse, *J. Phys. Chem. Lett.* **8**, 5, 966−974, 2017.

[2] M. Liebel, C. Schnedermann, T. Wende e P. Kukura, Principles and Applications of Broadband Impulsive Vibrational Spectroscopy, *J. Phys. Chem. A*, **119**, 9506−9517, 2015.

[3] F. Harris, On the Use of Windows for Harmonic Analysis with the discrete Fourier transform, *Proc. IEEE,* **66**, 51−83, 1978.

[4] R. Feynman, Slow Electrons in a Polar Crystal, Phys. Rev. **97**, 660 (1955).

[5] O. V. Selyugin, and M. A. Smondyrev, Phase Transition and Padé Approximants for Fröhlich Polarons, Phys. status solidi **155**, 155–167 (1989).

[6] A. S. Alexandrov and J. T. Devreese , Advances in Polaron Physics, Springer (2010).
